# Acupuncture for dyspnea in advanced cancer: a randomized, placebo-controlled pilot trial [ISRCTN89462491]

**DOI:** 10.1186/1472-684X-4-5

**Published:** 2005-08-18

**Authors:** Andrew J Vickers, Marc B Feinstein, Gary E Deng, Barrie R Cassileth

**Affiliations:** 1 Integrative Medicine Service, Memorial Sloan-Kettering Cancer Center, New York, USA; 2 Biostatistics Service, Memorial Sloan-Kettering Cancer Center, New York, USA; 3 Pulmonary Service, Memorial Sloan-Kettering Cancer Center, New York, USA

## Abstract

**Background:**

Dyspnea, or shortness of breath, is a common symptom in patients with advanced cancer. Pharmacologic management is of proven benefit, but it does not help all patients. Preliminary data suggest that acupuncture can relieve dyspnea in a variety of populations, including cancer patients. We conducted a pilot study (ISRCTN89462491) preparatory to a fully powered randomized, placebo-controlled trial to determine whether acupuncture reduces dyspnea in patients with lung or breast cancer.

**Methods:**

The study sample was comprised of forty-seven patients with lung or breast cancer presenting with dyspnea. Patients receiving symptomatic treatments were not excluded as long as no changes in management were planned during the trial. Patients were randomized to receive a single session of true or placebo acupuncture in addition to their existing dyspnea treatments. Semi-permanent acupuncture "studs" were then inserted: patients applied pressure to these studs twice a day to provide ongoing stimulation to acupuncture points. The subjective sensation of dyspnea was assessed with a 0 – 10 numerical rating scale immediately before and after acupuncture treatment and daily for a week thereafter.

**Results:**

All but two of 47 randomized patients provided follow-up data. Dyspnea scores were slightly higher for patients receiving true versus placebo acupuncture, for both the period immediately following acupuncture treatment and for the daily one week follow-up (differences between means of 0.34, 95% C.I. -0.33, 1.02 and 0.56, 95% C.I. -0.39, 1.51). The 95% confidence interval excludes the prespecified minimum clinically significant difference of a 20% greater improvement in dyspnea for patients receiving acupuncture.

**Conclusion:**

The acupuncture technique used in this trial is unlikely to have effects on dyspnea importantly larger than placebo for patients with advanced cancer.

## Background

Dyspnea, or shortness of breath, is defined as the subjective experience of difficulty breathing. It is a common symptom in cancer patients, particularly those with advanced cancer. For example, in a general survey of 100 outpatients and 140 inpatients at a Veterans' Affairs Medical Center, at least some dyspnea was reported by 50% of patients [1]. Studies of patients with advanced disease find that 50 –70% experience significant dyspnea[2,3]. Although dyspnea is most commonly associated with lung cancer[3], high rates also occur in patients with breast cancer with pulmonary metastases[4].

Dyspnea in cancer patients has numerous causes. It may result from complications of the cancer itself, such as pulmonary embolism, pleural effusion, anemia, and endobronchial obstruction or from conditions associated with a risk factor such as chronic obstructive pulmonary disease (COPD) in smokers. It may also result from disorders not directly attributable to the lungs, such as congestive heart failure or anemia. Treatment of these patients is typically guided by the identification of a specific underlying abnormality. Pleural effusions, for instance, are treated by thoracentesis, COPD is treated by bronchodilators and/or steroids, and anemia is treated by transfusion.

Unfortunately, a specific lung or cardiac pathology is not identifiable in approximately one-quarter of patients[2]. Moreover, dyspnea may result from causes in which definitive treatment has been unsuccessful or not practical. A number of interventions have been attempted for such patients, who often have advanced cancer within the lung parenchyma or endobronchial disease refractory to external beam radiation. Several randomized trials have shown that both opioids and supplemental oxygen can alleviate the subjective sensation of dyspnea among patients with advanced cancer.

For example, in a randomized, double-blind, crossover trial, dyspnea sensation fell by 25 mm on a 100 mm visual analog scale after morphine, with little change on placebo (p < 0.01 for difference between groups)[5]. Using a similar randomized, crossover design, Bruera et al. reported highly significant differences between periods when patients received oxygen as compared to periods when unsupplemented air was administered [6]. However, such measures are not effective in all patients, and the problem of dyspnea in the cancer patient is often frustratingly difficult to treat. A multinational study of end-of life-care found that dyspnea required sedation in 25 to 50% of patients during the last week of life[7]. It is for these difficult to treat cancer patients that additional therapeutic options are needed.

A number of randomized controlled trials have examined acupuncture for shortness of breath in patients without cancer. For example, a randomized, double-blind, placebo-controlled trial of acupuncture for methacholine-induced asthma reported significant differences in favor of acupuncture[8]. A similar study involved a standardized running test to induce bronchorestriction in asthmatics. Exercise-induced reductions in lung function were significantly lower in real compared to placebo acupuncture[9]. A trial in COPD reported significant differences between groups for subjective breathlessness. Thirty-one patients were randomized to self-administer finger pressure ("acupressure") at true or sham acupuncture points on a double-blind, crossover basis. Acupressure led to approximately a one-third reduction of scores on a VAS of dyspnea compared to about a 20% improvement in placebo controls[10].

Only one trial has been reported in cancer. Although uncontrolled, the results are provocative. Thirty cancer patients in palliative care received a single session of acupuncture. The mean visual analog score for breathlessness before acupuncture treatment was 42; this fell to 24 immediately following 10 minutes of needle insertion, an improvement that was maintained at six hour follow-up. Symptom scores returned to baseline 24 hours later[11].

Given these data, we believed it would be worthwhile to investigate whether cancer-related dyspnea can be relieved by acupuncture. Acupuncture is a complementary therapy, and therefore was administered as a supplement to use of pharmacologics or oxygen for dyspnea. Placebo control was employed because our endpoint was subjective and because acupuncture had not previously been found superior to placebo for cancer-related dyspnea. Our overall objective was to determine whether true acupuncture was more effective than placebo for alleviating dyspnea in patients with advanced lung or breast cancer. Here we report the results of a pilot study that aimed to refine the methodology for an anticipated definitive trial.

## Methods

Recruitment for the trial took place between July 2001 and March 2004 among patients under treatment for advanced lung or breast cancer at Memorial Sloan-Kettering Cancer Center (MSKCC). Patients age 18 or above with subjective complaint of shortness of breath and scoring grade two or higher on the American Thoracic Society Breathlessness Scale were eligible. Patients must have pursued a trial of steroid medication for dyspnea, if indicated, for at least 48 hours. Patients were excluded if any of the following applied: shortness of breath predated cancer diagnosis (e.g. asthma); recent onset of symptoms (< 7 days); anemia (defined as hemoglobin < 8 gm/dl); recent acupuncture; contraindications to acupuncture such as heart valve dysfunction or pancytopenia; planned *initiation *or *change *in oncologic therapy or symptomatic management of breathlessness (continuation of any existing management was allowed), or likelihood of patient death during the course of anticipated participation in the trial. Patients were also excluded if the primary cause of dyspnea was thought to be congestive heart failure, sarcoid disease, hypersensitivity pneumonitis, cryptogenic organizing pneumonitis, pneumothorax, chest wall deformity, obesity, neuromuscular disorders, pulmonary vascular disease, hepatomegaly or phrenic nerve paralysis syndrome. If the primary cause of dyspnea was ascites, effusion, pneumonia, large airway obstruction, superior vena cava syndrome or pulmonary embolism, patients were eligible only if they remained short of breath despite conventional therapy administered by their primary oncologist, or if they had refused such therapy.

Eligible consenting patients were randomized by telephone using the MSKCC clinical research database. Randomization used randomly permuted blocks with the cancer diagnosis (lung/breast) and breathless at rest (yes/no) as strata. The use of independent telephone registration and randomization ensured concealment of treatment allocation. Patients, researchers and others involved in patient care were blind to study group; only the acupuncturists and a researcher not associated with the study were aware of which patients received true and which placebo treatment.

Treatment consisted of two phases, acupuncture and acupressure. Patients received either true acupuncture followed by true acupressure, or placebo acupuncture followed by placebo acupressure. The first "acupuncture phase" consisted of a single treatment in which real or placebo needles were applied for 15 minutes at true or sham points, respectively. In the true acupuncture group, the needles used were stainless steel AsiaMed No.16 30 mm × 0.30 mm inserted to the traditional Chinese depth of 0.5 cm – 1.5 cm. Auricular points were needled with stainless steel Seirin D type No. 1 15 mm × 0.16 mm. As is common in traditional acupuncture, practitioners attempted to elicit *de qi *to help determine exact point location, but there was no manipulation of needles after placement. Placebo needles consist of a blunted needle that moves up inside its handle instead of into the skin. This technique has previously been demonstrated to be indistinguishable from true acupuncture[12]. Patients were told that in placebo acupuncture "the needles are placed so that they do not stimulate the correct acupuncture points. However, they do not look or feel any different from real needles." Immediately after insertion of the needles, patients were asked to assess the credibility of their treatment using a previously published scale[13,14].

The point prescription is described in Table 1. True points were chosen on the basis of the prior case series[11] and points traditionally used for breathlessness[15]; sham points were chosen in body areas away from true acupuncture points. The point prescription was modified slightly during the trial (details available on request), primarily to improve patient comfort. Patient outcome was not affected by the prescription used (see Results). Immediately after needle insertion, acupuncturists completed an audit sheet verifying the acupuncture points used. These records were routinely reviewed, and acupuncturists were found to have followed the acupuncture point prescription.

**Table 1:** Location of acupuncture points.

Point	Laterality	Anatomical location
Ren6	Unilateral	On the midline of the lower abdomen, 1.5 cun* inferior to the umbilicus and 3.5 cun* superior to the pubic symphysis
LU1	Bilateral	On the lateral aspect of the chest, in the first intercostal space, 6 cun* lateral to the midline,
LU7	Bilateral	On the radial aspect of the forearm, in the cleft between the tendons of brachioradialis and abductor pollicis longus
ST36	Bilateral	Below the knee, 3 cun* inferior to Dubi ST35, one finger breadth lateral to the anterior crest of the tibia.
KI6	Bilateral	1 cun* below the prominence of the medial malleolus, in the groove formed by two ligamentous bundles.
Auricular lung point	Bilateral	Medial and distal aspect of the tragus
Auricular kidney point	Bilateral	Medial and proximal aspect of the cymba conchae
Sternal points OR Ren17	Unilateral	Two needles inserted in the top two inches of the sternum and inserted down to the periosteum. Weak or cachectic patients were treated at Ren17, which is found on the anterior midline, at the level with the fourth intercostal space, midway between the nipples
Sham sternal	Unilateral	Two points anywhere in the upper two inches of the sternum: placebo needle only
Sham1	Bilateral	Posterior wrist, between radius & ulna, 1 cun* distal to radial & ulnar heads
Sham2	Bilateral	Anterior arm, 3 cun* proximal and 3 cun* medial to the antecubital crease
Sham3	Bilateral	Anterior arm, center of biceps brachii (midway between shoulder joint and antecubital crease)
Ear Sham	Bilateral	Adjacent to the "finger point", just below the helix of the auricle, at the apex

* a "cun" is a practical measurement used by acupuncturists equivalent to the greatest width of a patient's thumb at the distal phalanx

One hour after removal of needles, patients started the second "acupressure phase." Stainless steel AcuMedic acupressure studs, sometimes described as "press," "semi-permanent" or "intradermal" acupuncture needles, were used. These consist of a 2 mm × 0.28 mm acupuncture needle attached to a metal ring embedded in surgical tape. When the surgical tape is pressed on the skin, the needle pierces the skin but its diameter is so fine that this sensation is neither painful nor even immediately obvious. Acupressure studs have been used in a number of studies with cancer patients[16,17]. Placebo studs have no needle: they were specially designed for research in end-of-life populations by the author of the previous research on acupuncture for cancer related breathlessness[11].

To ensure retention of studs and guard against infection, the studs were covered with Tegaderm following application. Patients in the true acupuncture group were treated with true studs at ST36; Sternal points or Ren17; Auricular lung point and Auricular kidney point (see Table 1). Placebo patients received placebo studs at sham1, sham2 and ear sham. We thought it possible that patients might remove the studs before the end of the trial and that studs with a visible needle might be more credible than those without. Therefore, we also applied a placebo stud at sham1 to the acupuncture group and a true stud at sham3 to the placebo group. We told patients that some studs had small needles, others did not and that the choice depended on where they were placed on the body. Patients were also told that those in the placebo group received treatment at points not thought to help breathlessness.

Following application of the studs, patients were instructed to apply pressure to the study by making small circular movements with the fingers of the opposite hand, 2 – 3 cycles per second for 1 – 2 minutes per point. As is typical for self-administered acupressure, patients were encouraged to apply acupressure this way on waking, in the early afternoon and during any exacerbation of symptoms. Initial instruction was provided verbally, at which time patients were asked to confirm their understanding by demonstrating the procedure. Patients also were given easy-to-read written materials describing the acupressure procedure.

Study acupuncturists are certified by the National Certification Commission for Acupuncture and Oriental Medicine (NCCAOM) and are licensed to practice acupuncture in New York State. They have used acupuncture in clinical practice for 3 – 25 years. All were employed at MSKCC during the study and had considerable experience in treating cancer patients.

Outcome was measured in two ways. Every 15 minutes for 75 minutes immediately before acupuncture treatment and one hour immediately after, patients rated their current level of breathlessness on a 0 to 10 rating scale. They then completed a dairy daily for seven days, recording their average level of breathlessness through the day with the same 0 to 10 scale, and recorded compliance with acupressure.

The protocol was modified after 16 patients were accrued, as we found that many in-patients had been excluded because changes in management were likely in the subsequent seven days. We therefore modified the protocol so that inpatients did not take part in the acupressure phase. Moreover, many outpatients who complained of breathlessness in everyday life did not record significant breathlessness during the waiting period immediately before acupuncture. Accordingly, we amended the protocol so that outpatients completed the daily breathlessness diary for a week at baseline. Only patients scoring a mean of two or more were eligible. Out-patients who reported no breathlessness at rest were not asked to report on symptoms in the period immediately before and after acupuncture.

Preliminary power calculations on the basis of published data[10,11] suggested that a sample of 120–150 patients would be required to provide power to detect a clinically significant difference between groups, defined as a 20% lower follow-up dyspnea score in the acupuncture group compared to placebo. For this pilot we sought a sample of 40–50 patients. We felt that a sample of this size would give us sufficient methodologic experience to conduct an adequate fully powered study. Comparisons between groups were by analysis of covariance (ANCOVA) with baseline score and randomization strata as covariates. Baseline score for the acupressure phase was the one week baseline diary, if available, otherwise the immediate pre-treatment breathlessness score was used. Patients were analyzed in their randomized groups regardless of treatment received. Prespecified sensitivity analyses were to examine the effects of credibility on outcome and to assess outcome for the acupuncture phase only in patients with pre-treatment dyspnea scores greater than two. Statistical analysis was conducted using Stata 8 (College Station, Texas). No interim analyses were planned and the data were not analyzed before study closure.

The study was approved by the institutional review board at MSKCC in accordance with an assurance filed with and approved by the Department of Health and Human Services. Written informed consent was obtained from each participant.

## Results

**Figure 1:**
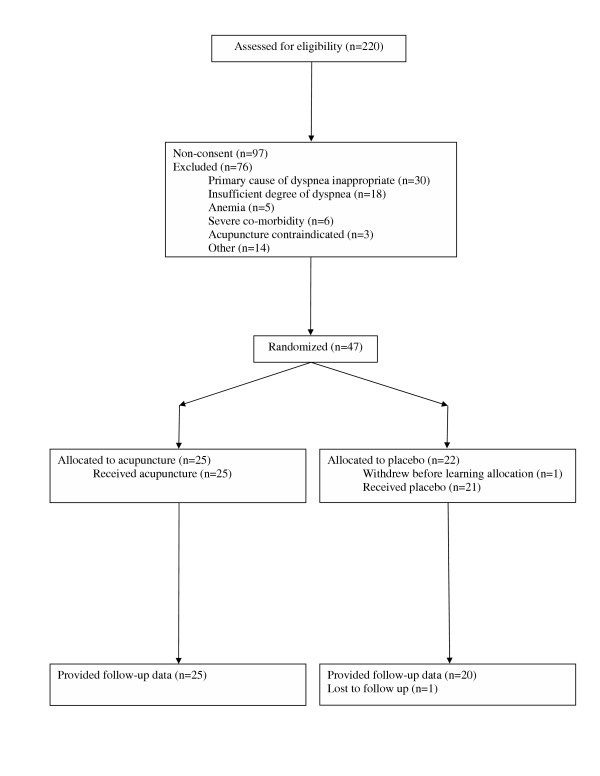
Flow of participants through the trial.

Flow of participants through the trial is shown in figure 1. Raw data are provided in Additional file 1. Table 2 shows data on study completers. Groups are balanced for baseline characteristics such as age, sex and diagnosis, and for postrandomization characteristics such as credibility and compliance. The similarity among credibility scores suggests that blinding was maintained, a conclusion also supported by analysis of research assistant notes: of the 21 patients who commented on allocation during post-study debriefing, 12 claimed to be unaware of allocation and only four of the remaining nine made a correct guess as to treatment received. Use of steroids, slightly more common in the placebo group, was not a strong predictor of outcome and there was no interaction between steroid use and group allocation. No patient changed use or dose of diuretics, opiates, bronchodilators or steroids during study participation.

**Table 2:** Baseline characteristics of patients providing post randomization data. Values are number (percentage) or mean (standard deviation)

	Acupuncture n = 25	Placebo n = 20
Female	15 (60%)	13 (65%)
Age	63.0 (12.8)	67.0 (11.4)
Diagnosis		
breast	5 (20%)	4 (20%)
lung	20 (80%)	16 (80%)
Breathless at rest	9 (36%)	6 (30%)
Credibility score*	2.24 (0.46)	2.10 (0.59)
Steroid use:**		
None	15 (60%)	9 (47%)
<40 mg prednisone equivalents	6 (24%)	8 (42%)
40+ mg prednisone equivalents	4 (16%)	2 (11%)
Diuretics**	3 (12%)	0 (0%)
Opiates**	5 (20%)	3 (16%)
Bronchodilators**	12 (48%)	7 (37%)
Fully compliant with acupressure+	8 (80%)°	6 (75%)°°

* two missing observations in acupuncture group; three missing observations in placebo group

** one missing observation in placebo group

^+ ^n = 10 acupuncture, n = 8 placebo

° one patient completely non-compliant, one patient 85% compliant

°° one patient 33% compliant, one patient 85% compliant

Breathlessness scores are reported in Table 3. We give results separately for dyspnea measured immediately after acupuncture, and for dyspnea assessed by daily diary. Some patients reporting dyspnea scores immediately after acupuncture treatment had negligible pre-treatment dyspnea. Hence we also provide a sub-group analysis of the immediate post-treatment scores, excluding patients scoring a mean of 2 or below. For all analyses, patients receiving true acupuncture reported slightly higher scores at follow-up, approximately equivalent to 10% of baseline. Patients in both groups improved, but no important differences between groups emerged. The upper bound of the 95% confidence interval for the difference between means excludes our prespecified minimum clinically significant difference of a 20% greater improvement in dyspnea for patients receiving acupuncture. Credibility did not predict breathlessness scores, either in the immediate post-treatment period (p = 0.4) or during the one week of diary recording (p = 0.9). Moreover, including credibility in the model for treatment effect did not importantly modify estimates.

**Table 3:** Breathlessness scores. Baseline scores only given for patients providing relevant follow-up data. Values are mean (standard deviation)

Endpoint	Group	Baseline	Follow-up	p value for within group change	Difference between means^+^	95% C.I.	p value for between group comparison
Immediately post-treatment	Acupuncture	n = 19: 4.09 (2.32)	n = 19: 3.36 (2.21)	0.003	0.34	-0.33, 1.02	0.3
	Placebo	n = 14: 3.41 (2.79)	n = 14: 2.42 (2.64)	0.003			
Immediately post-treatment (pre-treatment score >2)	Acupuncture	n = 15: 4.87 (1.92)	n = 15: 3.99 (2.03)	0.003	0.45	-0.55, 1.46	0.4
	Placebo	n = 8: 5.28 (2.18)	n = 8: 3.92 (2.50)	0.01			
Mean of seven day breathlessness diary	Acupuncture	n = 10*: 6.58 (1.71)	n = 16*: 5.07 (2.12)	0.4	0.56**	-0.39, 1.51	0.2
	Placebo	n = 7*: 5.99 (1.71)	n = 14*: 3.77 (2.39)	0.07			

^+ ^Positive indicates worse score in acupuncture group.

* Some patients providing post-treatment diaries gave baseline data only for the immediately pre-treatment period

**n = 13 for placebo group: one patient had no baseline data.

We conducted exploratory analyses to determine whether acupuncture prescription affected outcome. Outcome did not differ by point prescription in either the subgroup analysis (Table 4) or by including prescription and prescription by group interaction in the ANCOVA model.

**Table 4:** Effect of acupuncture point prescription on outcome.

Endpoint	Point prescription	n	Difference between groups^+^	95% C.I.
Immediately post-treatment	Initial	15	-0.23	-1.23, 0.77
	Modified	18	0.87	-0.19, 1.94
Daily Breathlessness diary	Initial	12	0.63	-0.54, 1.79
	Modified	17	0.43	-0.92, 1.77

^+ ^Positive indicates worse score in acupuncture group

No adverse events were causally related to acupuncture treatment.

## Discussion

Although intended as a methodologic pilot, our results are unexpectedly precise enough to warrant clinical recommendations. The confidence interval for the difference between groups is sufficiently narrow for us to conclude that, even in the best case scenario, the acupuncture technique tested is not importantly superior to placebo for dyspnea in cancer patients. Based on our clinical judgment of how much improvement we should expect to make worthwhile the time and trouble associated with acupuncture, we pre-specified a 20% difference between groups as clinically meaningful. It is against this criterion that we draw our conclusion of "no effect". Yet we can exclude even smaller levels of benefit, such as 10%. We find it unlikely that any patient or clinician would deem acupuncture to be worthwhile for a benefit of one third of a point on a 0 – 10 scale.

There are several possible limitations of our study. First, protocol changes concerning intervention, eligibility and evaluation, although common for a methodologic pilot, are not ideal when drawing conclusions about the effectiveness of a treatment. Nonetheless, we feel that these protocol changes did not have an important influence on our results. Changes in the methods of evaluation were designed to reduce drop-out and improve the measurement of baseline symptoms. These changes should increase the precision of results. Amendments to eligibility requirements were made to ensure that all patients had non-zero symptom scores at baseline and thus ensure that the trial included only those for whom benefit was measurable. Modification of the intervention itself is potentially more problematic. Most of the acupuncture point prescription changes were made to increase patient comfort. For example, we initially used the PC6 point on the wrist, but patients complained that studs inserted there caused radiating pain, apparently due to the proximity of PC6 to the median nerve. If our initial, but not modified, prescription were of value, it would be clinically irrelevant as an effective but intolerable treatment has little clinical role. Moreover, we saw no evidence that outcome was affected by the particular prescription used.

Patients in this study received only a single acupuncture treatment. This does not reflect traditional acupuncture practice, and it is possible that repeated treatment might have a cumulative effect. Our study design was based on prior research that suggested immediate effects on dyspnea following a single session of acupuncture[8,11] Delayed or cumulative effects are of questionable relevance for symptomatic management in patients with late stage cancer. Moreover, daily acupressure treatment had no apparent effect.

It is possible that more encouraging findings may have emerged had we used a different acupuncture technique, such as modifying the point prescriptions for each patient. Our study population reflected a typical spectrum of cases requiring symptomatic management of dyspnea, and was thus rather heterogeneous. Therefore, the possibility that acupuncture might be effective for dyspnea in a sub-group of patients with a particular pathology cannot be ruled out. There is a dearth of systematically collected data to inform decisions about study design with respect to either the most effective acupuncture technique or the most responsive patient group.

Our study provides some evidence of a placebo effect. Symptom scores fell by approximately 20% immediately after acupuncture in both groups (p = 0.003), a result unlikely to be explained by regression to the mean or other effects. It is plausible that such a result would have been considered "positive" in a single-arm study, underscoring the importance of randomized trials for evaluating complementary therapies used to treat cancer-related symptoms.

Accrual to the study was relatively slow, with only approximately 15 patients entering per year. This is due in part to the fact that MSKCC does not provide hospice services, which limits the number of end of life patients, when dyspnea is most prevalent. Accrual was also limited by patient refusal, as nearly half of patients approached declined participation. Patients with advanced cancer admitted to MSKCC generally have severe medical problems. Accordingly, they often feel overwhelmed, and a clinical trial, regardless of its perceived benefits and risks, is perceived as an additional burden. Patients commonly noted in response to the invitation to participate that "I just can't face dealing with this right now".

## Conclusion

We conclude that the acupuncture technique used in this trial is unlikely to have effects importantly larger than placebo for dyspnea in patients with advanced cancer.

## Abbreviations

ANCOVA: analysis of covariance

COPD: chronic obstructive pulmonary disease

VAS: visual analog scale

MSKCC: Memorial Sloan-Kettering Cancer Center

## Competing interests

The author(s) declare that they have no competing interests

## Authors' contributions

AJV, BRC and MBF designed the study. AJV analyzed data and wrote the manuscript. GED gave input to the analysis and manuscript with respect to the interpretation of the findings.

## Pre-publication history

The pre-publication history for this paper can be accessed here:



## Supplementary Material

Additional File 1 This is an Excel file with raw data from the study. The first worksheet contains the data; the second, a list of variable descriptors. Click here for file
